# Supplementary material for: Multi‐dataset identification of innovative feature genes and molecular mechanisms in keratoconus
Source: J Cell Mol Med. 2024 Sep 19;28(18):e70079. doi: 10.1111/jcmm.70079 (PMC11412914; doi:10.1111/jcmm.70079)
Supplement: Supplementary file 2 — Material S2. [file JCMM-28-e70079-s002.zip › 7.GSEA analysis/5.RNF217-AS1/KEGG_PYRUVATE_METABOLISM.html]

Details for gene set KEGG\_PYRUVATE\_METABOLISM[GSEA]

|  || Dataset | RNF217-AS1.group.cls#h\_versus\_l |
| Phenotype | group.cls#h\_versus\_l |
| Upregulated in class | l |
| GeneSet | KEGG\_PYRUVATE\_METABOLISM |
| Enrichment Score (ES) | -0.3362415 |
| Normalized Enrichment Score (NES) | -1.3339199 |
| Nominal p-value | 0.16237624 |
| FDR q-value | 0.37030908 |
| FWER p-Value | 0.931 |
Table: GSEA Results Summary

  

Fig 1: Enrichment plot: KEGG\_PYRUVATE\_METABOLISM      
 Profile of the Running ES Score & Positions of GeneSet Members on the Rank Ordered List

  

| SYMBOL | TITLE | RANK IN GENE LIST | RANK METRIC SCORE | RUNNING ES | CORE ENRICHMENT || 1 | LDHAL6A | na | 6613 | 0.164 | -0.1402 | No |
| 2 | ME3 | na | 7026 | 0.152 | -0.1053 | No |
| 3 | PDHA1 | na | 8282 | 0.121 | -0.1043 | No |
| 4 | ACOT12 | na | 10511 | 0.078 | -0.1446 | No |
| 5 | PDHA2 | na | 12139 | 0.053 | -0.1751 | No |
| 6 | HAGHL | na | 12598 | 0.047 | -0.1737 | No |
| 7 | PC | na | 12786 | 0.045 | -0.1653 | No |
| 8 | ME2 | na | 13171 | 0.040 | -0.1642 | No |
| 9 | ACYP2 | na | 15415 | 0.015 | -0.2243 | No |
| 10 | LDHA | na | 16042 | 0.008 | -0.2398 | No |
| 11 | PKLR | na | 16050 | 0.008 | -0.2375 | No |
| 12 | LDHB | na | 16493 | 0.004 | -0.2491 | No |
| 13 | ACSS2 | na | 16840 | 0.000 | -0.2590 | No |
| 14 | ACAT2 | na | 17362 | -0.005 | -0.2725 | No |
| 15 | ACACA | na | 18261 | -0.013 | -0.2943 | No |
| 16 | GRHPR | na | 19117 | -0.021 | -0.3125 | No |
| 17 | PDHB | na | 19823 | -0.028 | -0.3241 | No |
| 18 | ALDH3A2 | na | 20121 | -0.031 | -0.3231 | No |
| 19 | GLO1 | na | 20577 | -0.036 | -0.3253 | Yes |
| 20 | ME1 | na | 20782 | -0.038 | -0.3196 | Yes |
| 21 | MDH2 | na | 20928 | -0.039 | -0.3117 | Yes |
| 22 | HAGH | na | 20981 | -0.040 | -0.3010 | Yes |
| 23 | MDH1 | na | 21164 | -0.042 | -0.2935 | Yes |
| 24 | ACAT1 | na | 22576 | -0.057 | -0.3167 | Yes |
| 25 | ACSS1 | na | 22928 | -0.061 | -0.3081 | Yes |
| 26 | ALDH9A1 | na | 23254 | -0.065 | -0.2976 | Yes |
| 27 | LDHD | na | 24022 | -0.076 | -0.2965 | Yes |
| 28 | PKM | na | 25041 | -0.091 | -0.2979 | Yes |
| 29 | ALDH7A1 | na | 25259 | -0.095 | -0.2751 | Yes |
| 30 | LDHC | na | 25553 | -0.099 | -0.2531 | Yes |
| 31 | DLD | na | 25598 | -0.100 | -0.2236 | Yes |
| 32 | ACYP1 | na | 25738 | -0.102 | -0.1962 | Yes |
| 33 | LDHAL6B | na | 26559 | -0.117 | -0.1839 | Yes |
| 34 | DLAT | na | 26739 | -0.121 | -0.1520 | Yes |
| 35 | PCK1 | na | 27189 | -0.130 | -0.1249 | Yes |
| 36 | ACACB | na | 27956 | -0.147 | -0.1018 | Yes |
| 37 | AKR1B1 | na | 28116 | -0.152 | -0.0598 | Yes |
| 38 | ALDH1B1 | na | 28642 | -0.166 | -0.0239 | Yes |
| 39 | PCK2 | na | 30569 | -0.226 | -0.0101 | Yes |
| 40 | ALDH2 | na | 33631 | -0.423 | 0.0316 | Yes |
Table: GSEA details [plain text format]

  

Fig 2: KEGG\_PYRUVATE\_METABOLISM      
 Blue-Pink O' Gram in the Space of the Analyzed GeneSet

  

Fig 3: KEGG\_PYRUVATE\_METABOLISM: Random ES distribution      
 Gene set null distribution of ES for **KEGG\_PYRUVATE\_METABOLISM**
